# Downregulation of the H-2K^d^ gene by siRNA affects the cytotoxicity of murine LAK cells

**DOI:** 10.1186/1475-2867-13-112

**Published:** 2013-11-09

**Authors:** Xin Liu, Xin Cui, Ningning Shan, Ying Li, Xiaosheng Fang, Mei Ding, Xin Wang

**Affiliations:** 1 Department of Hematology, Shandong Provincial Hospital Affiliated to Shandong University, 324 Jing Wu Rd, Jinan, Shandong 250021, China

**Keywords:** RNA interference, siRNA, Posttranslational gene silencing, Lymphokine-activated killer

## Abstract

To investigate the effect of the H-2K^d^ gene on the lymphocyte membrane, we constructed a small interfering RNA (siRNA) that targets the H-2K^d^ gene and compared the cytotoxicity of mouse lymphokine-activated killer (LAK) cells with different H-2K^d^ expression states. H-2K^d^-targeting siRNA was transfected into spleen lymphocytes of BALB/C mice. Flow cytometry (FCM) was then performed to examine the expression of the H-2K^d^ gene in the transfected and control cells. Additionally, the cytotoxicity of the transfected cells toward the H22 and K562 cell lines was evaluated in vitro using the LDH release assay. H-2K^d^-targeting siRNA significantly reduced the expression levels of the target protein, whereas pure transMessenger and non-silencing siRNA did not inhibit H-2K^d^ expression at the concentrations tested. The cytotoxicity of siRNA-treated LAK cells toward H22 and K562 cells was reduced significantly. The knockdown of H-2K^d^ gene expression by siRNA may be associated with LAK cell cytotoxicity toward neoplasm cell lines.

## Introduction

Classical MHC-I molecules function both as alloantigens to trigger immune recognition and the rejection of allogeneic grafts in unmatched transplant recipients and as a platform to present self or foreign peptides that can be recognized by CD8+ T cells bearing a clonotypic T cell receptor [1]. It is well known that the MHC gene expression of tumor cells is down-regulated during malignancy [2-4]. Our previous investigations showed that the expression of host MHC is also down-regulated in tumor patients [5-8]. To more precisely investigate the functional relationship between host MHC-I molecules and tumor immunity, small interfering RNA (siRNA) duplexes were transfected into spleen lymphocytes of BALB/C mice to demonstrate the effects of MHC-I.

Posttranscriptional suppression of gene expression can be achieved by the introduction of sequence-specific siRNA [9,10]. Using this system, we achieved simultaneous down-regulation of the expression of MHC-I (H-2K^d^) genes in cultured lymphokine-activated killer (LAK) cells of BALB/C mice and the inhibition of the expression of endogenous MHC-I, leading to a reduction in the cytotoxicity of murine LAK cells. This reduction in cytotoxicity might be applied to determine the effect of H-2K^d^ on lymphocyte membranes in BALB/C mice.

## Materials and methods

### Cell culture

BALB/C mice were purchased from the Animal Laboratory Center of Shandong University, Jinan, China. LAK cells were generated from spleen cells obtained from BALB/C mice by culturing the spleen cells with IL-2 (1000 U/ml) for 36 h in 12-well culture plates until transfection using siRNA or transMessenger. The RPMI-1640 culture medium for the cell lines and lymphocytes contained 25 mM Hepes buffered with 44 mM NaHCO_3_ and supplemented with 10% fetal bovine serum (FBS). Cells were cultured in a humidified atmosphere containing 5% CO_2_ at 37°C. Additionally, the medium was supplemented with penicillin (100 U/ml) and streptomycin (100 μg/ml).

### Preparation and transfection of siRNA targeting the H-2K^d^ gene

In this study, we prepared siRNAs targeting the mouse H-2K^d^ gene. siRNAs was designed according to the method described by Elbashir [9,11]. Twenty-one-nucleotide RNAs (siRNA-1) and eighteen-nucleotide RNAs (siRNA-2) were chemically synthesized by QIAGEN (Germany). The siRNAs used in this study contained 3-overhangs of 2-deoxythymidine. The sequences of siRNA-1 pairs corresponded to nucleotides 264–282 and siRNA-2 corresponded to 427–445 after the start codon and were as follows:

Targeted sequence: AAGAGCGATGAGCAGTGGTTC

Sense strand: 5’-GAGCGAUGAGCAGUGGUUCdTdT-3’

Antisense strand 3’-dTdTCUCGCUACUCGUCACCAAG-5’

Targeted sequence: GGTGATCTCTGGCTGTGAA

Sense strand 5’- dTdTGGUGAUCUCUGGCUGUG--3’

Antisense strand: 3’-CCACUAGAGACCGACACdTdT-5’

For the preparation of duplexes, 20 μM mixed siRNAs were annealed in 1 ml of sterile buffer (100 mM potassium acetate, 30 mM HEPES-KOH, 2 mM magnesium acetate, pH 7.4) for 1 min at 90°C followed by a 1 h incubation at 37°C. Transfection of duplex siRNAs was performed according to the manufacturer’s instructions.

Annealed siRNA-1 and siRNA-2 were transfected into spleen lymphocytes that were in good condition, and the lymphocytes were seeded 36 h before transfection(siRNA/TransMessenger 1.6 μg/8 μl). Eighty-four hours after transfection, LAK cell proliferation was measured using the MTT colorimetric assay, and the effectiveness of the knockdown was assessed by flow cytometry. Cells in the non-silencing group were transfected with a non-silencing siRNA using transMessenger, and cells of the mock-transfected group were transfected with transMessenger only after culturing with IL-2 (1000 U/ml) for 36 h.

### Proliferative assay

Measurements of LAK cell proliferation were performed using the MTT (3-(4,5-dimethylthiazol-2-yl)-2,5-di-phenyltetrazolium bromide) colorimetric assay. Eighty-four hours after transfection (siRNA/TransMessenger 1.6 μg/8 μl, non-silencing siRNA or transMessenger only), LAK cells at 1 × 10^6^ cells/ml were plated in 96-well microtiter plates (Corning Costar, Cambridge, MA, USA) and incubated with 0.25 mg/ml MTT for 4 h at 37°C. Before the end of the assay, 100 μl of DMSO was added to each well. The amount of MTT formazan product was determined by measuring the absorbance (A) using a microplate reader at a test wavelength of 570 nm and a reference wavelength of 655 nm.

### Flow cytometry analysis of H-2K^d^ expression

The analysis of surface immunofluorescence was performed using FACScan flow cytometry (Becton Dickinson, USA). LAK cells (1 × 10^6^ cells/ml) to be analyzed for immunofluorescence were incubated with a saturating amount (10 μl) of FITC-labeled monoclonal anti-H-2K^d^ (Becton Dickinson, USA) for 30 minutes at 37°C, washed twice in the diluent and resuspended in PBS containing 1% formaldehyde and 0.5% sodium azide. Non-specific binding was subtracted using appropriate controls.

### Cytotoxicity assay

The target cells were placed in 96-well plates at 10,000 cells/well with the appropriate number of LAK cells as indicated in 0.2 ml of complete medium. After 4 h of incubation at 37°C in a humidified atmosphere containing 5% CO_2_, the LDH assay was applied according to the manufacturer’s instructions for the CytoTox 96^®^ Non-Radioactive Cytotoxicity Assay. The percentage-specific release was calculated as follows:

$$\%\;\text{Cytotoxicity}=\frac{\text{Experimental}-\text{Effector}\;\text{Spontaneous}-\text{Target}\;\text{Spontaneous}}{\text{Target}\;\text{Maximum}-\text{Target}\;\text{Spontaneous}}\times 100$$

Experimental counts were determined from triplicate wells.

### Statistical analysis

Data are presented as the means ± SEM. Statistical analysis was performed by one-way analysis of variance (ANOVA). All tests were performed using SPSS (version 16.0; SPSS, Inc., Chicago, IL, USA), and the level of significance was set at a *P* value less than 0.01.

## Results

### siRNA has no toxic effect on spleen LAK cells

No cell morphology abnormalities or cell breakage was observed in any group using an inverted microscope. Table 1 shows that the siRNA and transMessenger had no inhibitory effect on the proliferation of LAK cells.

**Table 1:** Analysis of the LAK activities of the different groups (n = 5)

**Experimental group**	**siRNA/ TransMessenger (μg/μl)**	**A (absorbance)**	**Compared with controls**
Control	0/0	1.46 ± 0.14	
siRNA	1.6/8	1.46 ± 0.09	*P* > 0.05
Mock-transfected	0/8	1.50 ± 0.13	*P* > 0.05
Non-silencing	1.6(Non-si)/8	1.48 ± 0.20	*P* > 0.05

### H-2K^d^ Expression in spleen LAK cells

**Figure 1:**
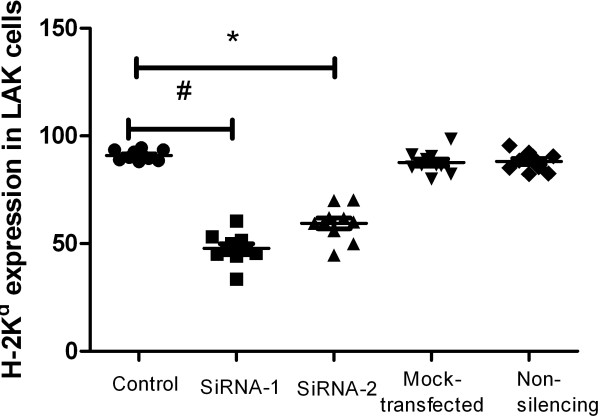
**Expression of H-2K**^**d **^**in spleen LAK cells from different groups.** Four days after cells were transfected with siRNAs, FCM was used to examine the expression of the target protein. H-2K^d^ expression in LAK cells were reduced by H-2K^d^ siRNA-1 and siRNA-2 compared with the level in control cells (#P < 0.05). No difference in H-2K^d^ expression in LAK cells was observed among the mock-transfected, non-silencing siRNA-transfected and control groups.

FCM was used to examine the targeted protein expression. Eighty-four hours after transfection, H-2K^d^ expression in LAK cells was reduced by H-2K^d^ siRNA-1 (47.8 ± 6.9%) and (59.5 ± 7.9%) by H-2K^d^ siRNA-2 compared with the levels in mock-transfected cells (87.7 ± 5.1%; *P* < 0.01), non-silencing siRNA-transfected cells (88.2 ± 4.3%; *P* < 0.01) and control cells (90.9 ± 2.4%; *P* < 0.01). No significant difference in H-2K^d^ expression in LAK cells was found among the mock-transfected, non-silencing siRNA-transfected and control groups (*P* > 0.05) (Figure 1).

### Calculation of LAK cell cytotoxicities

After induction with IL-2 for 5 days, LAK cells from BALB/C mice showed marked cytotoxicity toward H22 and K562 cells. The addition of siRNA-1-treated LAK cells resulted in a cytotoxicity decrease of 20.3% (P <0.05; effector/target cell ratio: 40:1; Table 2) toward H22 cells. Similar results were obtained in cultures of K562 cells, in which siRNA-treated LAK cells (effector/target cell ratio: 40:1) exhibited a decreased in cytotoxicity from (76.8 ± 5.8) to (49.6 ± 7.7) (P <0.05; Table 3). However, no significant difference was found among the mock-transfected, non-silencing siRNA-transfected and control groups in the present study.

**Table 2:** Analysis of LAK cytotoxic activity toward H22 cells (mean ± SEM, n = 8)

**Experimental group**	**Effector/target cell ratio**			
	**10:1**	**20:1**	**30:1**	**40:1**
Control	28.6 ± 4.1	45.2 ± 4.8	68.4 ± 5.0	81.6 ± 5.9
siRNA-1	27.6 ± 2.1	40.6 ± 5.1*#▲	55.4 ± 4.1*#▲	65.0 ± 2.1*#▲
Mock-transfected	26.9 ± 5.6	46.9 ± 5.7	70.3 ± 6.0	80.5 ± 4.6
Non-silencing	30.3 ± 3.9	44.3 ± 3.8	67.0 ± 4.9	82.0 ± 2.6

^*^P < 0.01, siRNA-transfected group compared with the control group;

^#^*P* < 0.01, siRNA-transfected group compared with the mock-transfected group;

^▲^*P* < 0.01, siRNA-transfected group compared with the non-silencing group.

**Table 3:** Analysis of LAK cytotoxic activity toward K562 cells (mean ± SEM, n = 8)

**Experimental group**	**Effector/target cell ratio**			
	**10:1**	**20:1**	**30:1**	**40:1**
Control	30.1 ± 9.8	50.1 ± 6.4	63.4 ± 7.6	76.8 ± 5.8
siRNA-1	28.4 ± 36.9*#▲	31.3 ± 6.5*#▲	36.9 ± 5.9*#▲	49.6 ± 7.7*#▲
Mock-transfected	28.9 ± 6.7	49.9 ± 5.8	66.3 ± 8.0	74.9 ± 8.1
Non-silencing	31.4 ± 8.7	51.2 ± 9.1	64.9 ± 8.9	80.0 ± 9.7

^*^P < 0.01, siRNA-1-transfected group compared with the control group;

^#^*P* < 0.01, siRNA-1-transfected group compared with the mock-transfected group;

^▲^*P* < 0.01, siRNA-1-transfected group compared with the non-silencing group.

## Discussion

The major histocompatibility complex (MHC) H-2K^d^, as the MHC-I molecule of BALB/c mice, is a cell surface glycoprotein that plays critical roles in the regulation of tumor immune responses. These molecules are expressed on the surface of all nucleated cells and are necessary for the presentation of peptide antigens to cytotoxic T-lymphocytes (CTLs) [12,13] and for the immune regulatory activity exerted by NK cells [14,15]. It is widely accepted that the total or partial loss of MHC class I molecules on tumor cells is one of the main mechanisms of tumor escape. However, the effects of MHC on peripheral blood mononuclear cells (PBMCs) remain unclear. In the present study, we first evaluated the cytotoxicity of LAK cells toward tumor cells with different expression states of MHC-I. Down-regulated MHC-I expression in LAK cells led to lower cytotoxicity toward H22 cells and K562 cells.

Studies have shown that the high-density expression of MHC class I molecules can protect T cells from deletion mediated by antibodies and macrophages [16]. When the expression of these MHC class I molecules is masked, this resistance collapses, indicating that highly expressed MHC class I molecules can prolong the survival time of T cells. Many experiments have also been designed to evaluate the significance of host MHC antigens [17-19]. Lemorvan [20] discovered that the expression of PBMCs HLA-B mRNA is reduced as one advances into old age, when one’s immune system is also weakened. Studies on varicella zoster virus (VZV) pathogenesis have demonstrated that cell surface MHC I expression is downregulated specifically in VZV-infected human CD3^+^ T lymphocytes [21,22]. siRNA was then applied to spleen LAK cells from BALB/C mice to assess the effects of MHC-I.

LAK cells are lymphocytes exposed to interleukin-2 for 4 to 6 days. Several studies have shown that the cellular population mediating LAK cell activity consists largely of IL-2-stimulated CTLs and NK cells. Specific surface markers have not been found on LAK cells [23,24]. The molecular mechanisms involved in the recognition of tumor cells by CTLs and NK cells have been partially elucidated in recent years. MHC class I-bearing cells interact with the T-cell receptor (TCR) on CD8+ CTL cells, triggering a cascade of T-signal events that ultimately lead to cell proliferation, cytokine production and target cell lysis [25-27]. This positive effect is associated with the expression of self-MHC-I-specific receptors on CTL and NK cells that were originally identified as inhibitory receptors in effector responses [28]. Several investigators have invoked a role for abnormally low expression of MHC class I molecules on tumors in the recognition process by correlating MHC-I expression with NK insensitivity utilizing IFN treatment, which increases MHC-I expression, or selection of cell clones with varying levels of MHC-I expression [29]. However, experiments utilizing transfection of MHC class I genes into sensitive cell lines have yielded conflicting results [30], indicating that MHC-I expression is not the sole recognition mechanism. The down-regulation of MHC-I antigens in NK and lymphocytes may provide an important mechanism in the tumor immune response. Large numbers of lymphocytes exist in the spleen in vivo; however, their slow proliferation prevents them from being cultured in vitro. Induced by IL-2, spleen lymphocytes signal to LAK cells, which can lyse not only self tumor cells but also isotype entity tumor cells [31,32]. Our study demonstrated that the inhibition of H-2K^d^ expression in mice reduced the cytotoxicity of LAK cells toward H22 and K562 target cells, suggesting that there is a direct correlation between H-2Kd expression and the cytotoxicity of CTLs and NK cells.

Our study strongly suggests that the resistance to tumors is influenced by MHC-I expression in the host as well as in the target cells. The expression of MHC molecules on the tumor cell surface is a feature of tumor cells, whereas the change in host MHC expression reflects the state of MHC in the whole body and represents the host’s immune nature. The expression of MHC-I molecules on host peripheral blood lymphocytes reflect their immune competence as an anti-viral and anti-tumor molecules. A decrease in host MHC-I expression, leading to the unresponsiveness of CTLs and NK cells due to the default of antigen presentation, may play an important role in tumor development. Measuring MHC-I expression will contribute to our understanding of host immune status and provide new insights into tumor immunity.

## Competing interests

The authors declared that they have no competing interests.

## Authors’ contributions

LX participated in experimental design and manuscript writing; CX contributed to data analysis; SN and LY contributed to experimental design and the statistical analysis; FXS and DM contributed to data analysis; WX contributed to experimental design and writing of the manuscript. All authors revised the manuscript critically and approved the final version to be published.
